# Burden of disease experienced by patients following a watch‐and‐wait policy for locally advanced rectal cancer: A qualitative study

**DOI:** 10.1111/codi.15838

**Published:** 2021-08-07

**Authors:** Alexander J. Pennings, Merel L. Kimman, Anke H. C. Gielen, Geerard L. Beets, Jarno Melenhorst, Stephanie O. Breukink

**Affiliations:** ^1^ Department of Surgery Maastricht University Medical Center Maastricht The Netherlands; ^2^ GROW School for Oncology and Developmental Biology Maastricht The Netherlands; ^3^ Clinical Epidemiology and Medical Technology Assessment Maastricht University Medical Center+ Maastricht The Netherlands; ^4^ Faculty of Health, Medicine and Life Sciences Maastricht University Maastricht The Netherlands; ^5^ Department of Surgery Netherlands Cancer Institute Amsterdam The Netherlands; ^6^ NUTRIM School of Nutrition and Translational Research in Metabolism Maastricht The Netherlands

**Keywords:** burden of disease, functional outcomes, interviews, patient reported outcomes, quality of life, rectal cancer, watch‐and‐wait

## Abstract

**Aim:**

Patient‐reported outcome measures (PROMs) are increasingly being used in routine cancer care to evaluate treatment and monitor symptoms, function and other aspects of quality of life (QoL). There is no suitable PROM for rectal cancer patients following a watch‐and‐wait (W&W) programme. Insight into patient experiences with this programme is an essential step in the development of a PROM. The aim of this qualitative study was to provide insights into the most important functional outcomes and QoL features experienced by patients during our W&W programme.

**Method:**

Patients with locally advanced rectal cancer who are enrolled in the W&W programme in the Netherlands were interviewed by telephone using a semistructured interview guide. All interviews were digitally audio‐recorded, transcribed verbatim and coded. A thematic approach was used to analyse the data and identify themes and subthemes of importance to patients.

**Results:**

Eighteen patients were interviewed (78% male, mean age 68 years, range 52–83 years). Physical complaints after treatment were present, most notably gastrointestinal problems, neuropathy and fatigue. Furthermore, patients were anxious about a possible recurrence, had a fear of surgery or a stoma, or were experiencing a general feeling of apprehension in daily life. Many patients had different coping mechanisms, such as acceptance, and there were few limitations in daily life.

**Conclusion:**

We identified important functional outcomes, such as gastrointestinal complaints, fatigue and neuropathy, in patients who were enrolled in this W&W programme. Furthermore, an emotional burden and unmet needs were reported by these patients. These findings can be used to improve clinical practice and inform the development of a PROM.

**What does this paper add to the literature?**

This is the first study dedicated to the experience of patients in a watch‐and‐wait programme for rectal cancer. The study provides new insights into the physical and emotional burden of patients in this programme; these insights can be used to develop a patient‐reported outcome measure to improve individualized care for this population.

## INTRODUCTION

In recent years, the organ‐preserving treatment method of a watch‐and‐wait (W&W) policy after a clinically complete response following neoadjuvant chemoradiation therapy (nCRT) has being increasingly adopted in the treatment of locally advanced rectal cancer [1, 2, 3, 4]. Its principle of having a clinically complete response to nCRT followed by a stringent follow‐up scheme rather than surgical treatment has proven its value, with a 5‐year overall survival of 85% and 5‐year disease‐specific survival of 94% [2]. Hupkens et al. state that patients enrolled in a W&W programme potentially have a better quality of life (QoL) and improved functional outcomes than patients who underwent nCRT and total mesorectal excision (TME) [5]. However, despite not undergoing surgery, approximately a third of the W&W patients experience symptoms of major low anterior resection syndrome (LARS) as a consequence of nCRT [5]. Nevertheless, the sample size in this and other studies is small, and the lack of validated instruments for the W&W population limits the generation of evidence [1, 6, 7]. During our W&W programme patients follow an intensive follow‐up schedule, consisting of digital rectal examination, MRI, endoscopy (with biopsy) and carcinoembryonic antigen measurements every 3 months for the first 2 years and 6‐monthly thereafter up to 5 years. Annual CT scans are used to detect distant metastases. During follow‐up it is important not only to focus on oncological outcomes and physical symptoms but also to monitor the emotional, social and cognitive health of patients. However, patients do not always discuss these outcomes with clinicians, and clinicians often underestimate how much importance patients attach to functional outcomes and QoL compared with survival [8, 9, 10].

Patient‐reported outcome measures (PROMs) reflect a patient’s own evaluation of his or her symptoms, functional outcome and QoL. The routine use of PROMs is reported to have a positive impact on the level of care and patient outcomes [11, 12, 13]. They serve as a foundation for patients to discuss with their healthcare workers the issues that are important to them, improving communication and shared decision‐making. PROMs may also encourage patients to reflect on their condition and to get a deeper understanding of how their condition affects them in their daily lives [11, 12, 13].

Questionnaires such as the EORTC‐QLQ‐C30 and EORTC QLQ‐CR38 are examples of PROMs developed for cancer patients [14, 15]. In addition, the Short Form 36 (SF‐36) [16], the Vaizey score [17], the LARS score [18], the IIEF and FSFI questionnaires to indicate sexual problems [19, 20] and the IPSS to assess problems of the urinary tract have been used in W&W patients [21]. None of these PROMs focus specifically on W&W patients. They were all developed and validated in patients who had surgery, except for the SF‐36 and EORTC‐QLQ‐C30 which are suitable for the general population and a wide range of cancer patients, respectively. As a result, the relevance and usefulness of these PROMs may be limited in W&W patients. For use in clinical practice, a short PROM would support follow‐up care as it would provide a clear overview of the problems experienced by W&W patients [22]. Currently, such a PROM is not available for patients enrolled in W&W programmes, and no data are available on patients’ experiences or outcomes during the W&W follow‐up period.

In this study, we aimed to identify the experiences and functional outcomes of W&W patients during their follow‐up period and how the disease, its treatment and the W&W programme affect their daily lives. Findings from this study can aid in the development of a PROM for W&W patients to be used in clinical care.

## METHOD

### Study design

This study had a qualitative research design with semistructured interviews to explore patients’ views regarding functional outcomes and QoL during our W&W programme. We chose semistructured interviews to ensure that certain topics were discussed while still allowing patients to discuss everything they wanted to share. A purposive sampling technique based on age, gender and time in follow‐up was used to include a variety of patients with a range of characteristics, representing the W&W population. The estimated sample size for the patient interviews was 15–20 patients, but saturation was used as a criterion for discontinuing the patient interviews [23].

All candidate patients were contacted by their treating colorectal surgeon (JM or SB) to assess their willingness to participate. If the patient was willing to participate, one researcher (AP) contacted the patient to explain the purpose of the study and procedures in depth, and to make an appointment for the interview. At the start of each interview written consent was obtained. The study protocol was approved by the Medical Ethical Committee of the Maastricht University Medical Center+, the Netherlands (METC‐2019‐1245). To ensure explicit and comprehensive reporting the Standards for Reporting Qualitative Research (SRQR) checklist was used [24].

### Study population

The study population consisted of patients with locally advanced rectal cancer who are enrolled now, or have been enrolled in the past, in our W&W programme as described by Maas et al. [1]. Exclusion criteria were insufficient proficiency in the Dutch language or recurrence of disease during follow‐up.

### Data collection

Between August 2019 and January 2020 a telephone interview was conducted with each patient. All interviews were conducted by the same researcher (AP) who is a male medical doctor working as a clinical investigator and trained in conducting interviews. The interviewer had no prior encounter or relationship with the patients. During the interviews, an interview guide was used that focused on three topics: (1) the initial treatment for rectal cancer, (2) experience during the W&W programme, (3) important functional outcomes according to the patient (see Appendix 1). The main focus of the interviews was on topics two and three, whereas topic one was used as an introduction to the interview. During each interview, patients were encouraged to describe the experiences and problems they had encountered during the W&W programme. The interview guide was designed to allow the patient to have the opportunity to elaborate on certain topics, so it contained follow‐up questions such as: ‘You mentioned [symptom/problem]. Can you tell me more about this?’ All interviews were digitally audio‐recorded and transcribed verbatim. Interview transcripts were fully anonymized.

### Data analysis

Two researchers (AP and AG) analysed the data manually using a thematic approach as described by Ritchie et al. [25]. The researchers independently read and reread the first five transcripts and coded them descriptively using an inductive approach, then they compared codes and resolved discrepancies to create a coding tree for the remaining transcripts. Any new codes were discussed and, if appropriate, added to the coding tree. Finally, all codes were analysed and similar concepts were grouped into (sub)themes. Themes were discussed with MK (a senior health technology assessment researcher, with a focus on outcomes research) and SB (a consultant in colorectal surgery at the Maastricht University Medical Center involved in the W&W research).

## RESULTS

Eighteen patients participated in the study, of whom 14 (78%) were male and 4 (22%) female with a median age of 68 years (range 52–83 years). All patients received nCRT according to the Dutch national colorectal cancer guidelines [26]. The mean follow‐up after inclusion into the W&W programme was 32 months (range 5–71 months). Patients had different lengths of follow up (nine patients ≤2 years, seven patients between 3 and 5 years and two patients >5 years) (Table 1).

**TABLE 1:** Patient characteristics

Characteristics	Individual interviews (*n* = 18)
Age (years), mean (range)	68 (52–83)
Male gender, *n* (%)	14 (78)
Duration of W&W (months), mean (range)	32 (5–71)
Completed W&W, *n* (%)	2 (11%)
Primary treatment, *n* (%)	
nCRT (capecitabine + radiotherapy)	15 (83.3)
nCRT + CAPOX	3 (16.7)
Clinical T‐stage^a^, *n* (%)	
cT2	3 (17)
cT3	14 (78)
cT4	1 (6)
Clinical N‐stage^a^, *n* (%)	
N1	13 (72)
N2	5 (28)

Abbreviations: *n*, number; nCRT, neoadjuvant chemoradiotherapy; W&W, watch‐and‐wait.

^a^ Clinical T‐ and N‐stage were acquired by biopsy and MRI.

Interviews lasted between 20 and 30 min. Analysis of the interviews showed that after the 15th interview no new codes emerged. However, the researchers conducted and analysed three additional interviews to ensure saturation, and still no new codes arose [23].

**FIGURE 1:**
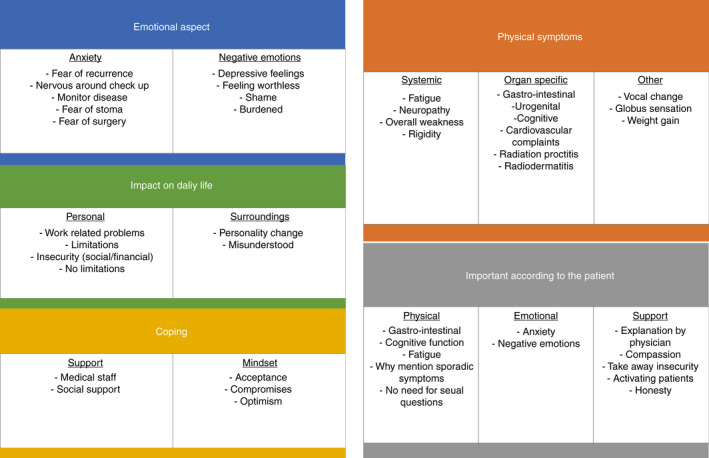
Schematic representation of themes and subthemes

Four main themes were identified in the interviews: physical symptoms, emotional aspects, coping and impact on daily life. Themes and associated subthemes are summarized in Figure 1.

### Physical symptoms

Patients experienced a range of symptoms during initial treatment with nCRT and the W&W programme. Gastrointestinal symptoms, prolonged fatigue and persistent neuropathy caused by chemotherapy were the most frequently mentioned symptoms during the W&W period. One patient mentioned: ‘As soon as I feel I have to go to the toilet I have to go, because I cannot defer defaecation for long.’ Many patients reported complaints of diarrhoea and pain with bowel movement during and shortly after initial treatment, but these complaints mostly disappeared within the first 6 months after treatment. Fatigue also seemed to diminish in patients who experienced it during the treatment period. Others, who did not notice it as much at the beginning, mentioned needing to rest during the day to be able to function in the evening. One patient said: ‘In the past (before treatment), I could sometimes take a nap in the afternoon, but now I have to do it every day otherwise I am exhausted at the start of the evening.’ Neuropathy was a more persistent complaint, still present in some patients 1 year after treatment without any indication of improvement. Cognitive problems such as concentration and memory problems were mostly mentioned by younger patients who had an active job at the time of the interview. Sexual dysfunction was experienced by some patients. The most common problem was erectile dysfunction expressed by male patients. The female patients in our study did not report problems related to sexual function. None of the patients mentioned urinary problems during follow‐up, but a few reported temporary problems during and shortly after CRT.

While most patients still experienced at least one physical complaint there were some patients who did not experience any physical complaints at the time of the interview. One patient stated: ‘I am as healthy as a horse.’

### Emotional aspects

The emotional impact of the W&W programme clearly emerged during the interviews. Anxiety was the most prominent emotion identified during the interviews, and was reported by the majority of participants. This included fear of recurrence, fear of potential surgery and a stoma, anxiety around check‐ups and a general feeling of apprehension in daily life. One patient explained ‘I think every cancer patient is scared that the cancer will come back.’ Another patient, when asked how he experienced the W&W programme, said: ‘It is a good experience as long as the results of the examination are positive.’ Patients also mentioned that a good result during follow‐up temporarily lowered their anxiety levels and as a new examination approached anxiety increased again. ‘You have to endure it and when you go home again you know that all is well and you can put it aside again.’ Some participants reported experiencing feelings of depression.

### Coping

A topic that emerged upon data analysis were the coping mechanisms of W&W patients. These coping mechanisms gave an insight into how the patients handled their physical and emotional difficulties. Putting their disease into perspective is something that almost all patients did. ‘I am lucky it ended well, my brother died of rectal cancer.’ Acceptance was often focused on the physical complaints they experienced and how these did not control their life in a significant way. Some patients related their symptoms to their age rather than to the disease: ‘You get older right, some things you just have to accept, I am not 25 anymore.’ During the W&W programme, patients felt supported by clinicians and properly informed about potential outcomes and their prospects for the future. Many felt they were listened to and that they could ask any questions about their treatment. ‘With my treating clinician I felt personally engaged and completely at ease.’ Furthermore, patients expressed that they received a lot of support from family and friends, which was beneficial for their mental wellbeing, especially during the initial treatment.

### Impact on daily life

When patients were asked what impact the diagnosis and the W&W programme had on their daily life, responses varied greatly. Patients did not feel limited in their daily life by the strict follow‐up schedule. In most cases, all investigations were scheduled on the same day, which was convenient and appreciated. Physical complaints as a result of the treatment were present, but only a minority still felt constrained by them in daily life. A few patients experienced work‐related problems, mostly due to cognitive impairment. As one patient mentioned: ‘I am a business advisor and you need to be able to express yourself well. I know exactly what I want to say but just cannot find the words.’ Patients felt that outsiders do not always understand the impact of the disease and treatment. ‘The outside world does not get it. Not everyone understands what the disease and treatments do to you.’ This feeling of being misunderstood affected their emotional wellbeing. Worries about social and financial insecurity due to the disease also emerged during the interviews. Nonetheless, some stated they did not feel any limitations to their daily life: ‘I do everything I did before the disease.’

Patients were asked if they felt satisfied that they had been included in the W&W programme. None of the patients regretted choosing the W&W programme. Based on the experience with the W&W programme and current knowledge, none of them would rather have opted for a surgical resection. The reason most expressed for this was that with the W&W programme they could avoid a stoma and that there was always the option to have surgery if the cancer returned.

### Patient‐relevant outcomes

In the final part of the interview, patients were asked what they felt were the important outcomes or issues to focus on during the W&W programme. The most dominant response was the emotional impact of being in a follow‐up programme, i.e. the uncertainty and fear of recurrence. The patients were positive about the communication by their physician about the disease, and the detailed explanation of endoscopy pictures and other results during the follow up consultations. Nevertheless, several patients pointed out that the potential emotional impact of the W&W programme does not get a lot of attention: ‘During the first two years there should be guidance by caregivers on how to deal with fear and the “waiting”.’ However, all patients expressed the view that physical function should be evaluated regularly during the W&W programme, and were happy with the attention given to this by clinicians.

## DISCUSSION AND CONCLUSIONS

This qualitative study showed that patients enrolled in our W&W programme experience physical symptoms such as gastrointestinal complaints, fatigue and neuropathy, both during and after the courses of treatment. Gastrointestinal complaints were the most often reported. There was also a clear emotional burden present after treatment and during the follow‐up period. Patients felt there was little attention given to emotional aspects specifically related to the W&W programme, for example how to deal with the waiting and fear of recurrence.

By including patients at different time points in their follow‐up, we were able to identify the range of issues that patients may face during their follow‐up. We found that patients’ perspectives may change over time. For example, we found that patients who were at least 2 years into the W&W programme reported fewer problems related to anxiety or insecurity compared with those who had just started within the programme.

We found that physical complaints were consistent with a recent study by Han et al. on symptoms after treatment for colorectal cancer [27]. Gastrointestinal complaints were the most common, most likely related to the effects of radiotherapy on anorectal function in this population [28]. Other studies confirm this unfavourable short‐ and long‐term effect of radiotherapy on anorectal function [29, 30]. Besides gastrointestinal symptoms, fatigue and neuropathy were common complaints that are frequently seen in colorectal cancer patients after chemo(radio)therapy [27]. Remarkably, no patients reported any urinary dysfunction, even though this problem is commonly reported after rectal cancer treatment, especially TME [31, 32, 33]. The fact that our patient population did not undergo surgery, thereby preserving pelvic innervation, could be an explanation for this finding. Furthermore, only a few male patients mentioned sexual dysfunction. This too could be related to the fact that our population did not undergo surgical resection. The absence of sexual dysfunction in the female patients was notable. Yet we cannot conclude that sexual dysfunction is absent or less important in female patients based on our study findings. Sexual dysfunction in women is multifactorial and complex, involving physical and psychological aspects such as libido, sexual arousal and body image [34]. It is possible that participants felt uncomfortable or the questions did not probe the participants to open up about such a sensitive and complex topic in our study setting (e.g. telephone interview, male interviewer). Furthermore, the small number of female participants may have resulted in underreporting of these problems. A recent study by Angenete et al. [35] showed that female rectal cancer patients indeed experience considerable sexual dysfunction in the form of dyspareunia and a reduced ability to achieve an orgasm after abdominoperineal resection. Nevertheless, women were less affected by these problems than men in their daily life.

Only a few patients reported cognitive impairment in daily life, mostly experienced during work‐related activities. However, a study by Vardy et al. [36] showed that colorectal cancer patients had significant cognitive impairment compared with healthy controls after neuropsychological testing. However, these results were only mildly associated with self‐reported cognitive impairment [36]. This could explain the low reports of cognitive impairment in this study.

The most prominent emotional impact after treatment and while in the W&W programme was anxiety in the form of fear of recurrence. This fear is common among cancer patients [37, 38]. Participants said that levels of anxiety increased around check‐ups. This may suggest that patients enrolled in the W&W programme experience more anxiety or anxiety‐related complaints than other CRC patients because of the strict follow‐up schedule. Compared with regular follow‐up in colorectal cancer, patients in the W&W programme undergo sigmoidoscopies and MRI scans every 3 months during the first 2 years. Hupkens et al. [5] conducted a study comparing W&W patients with patients who underwent nCRT and surgery (TME) and found that W&W patients scored lower on general health according to the SF‐36 questionnaire. The authors hypothesized that this might be because the rectum is not resected and the possibility of regrowth still exists [5]. However, participants in this study stated that a good result during follow‐up visits reassured them that no tumour was present and immediately decreased their fear. Patients are informed at the start of the W&W programme, however, that the probability of recurrence is highest during the first 2 years after treatment [39]. While there is no difference in overall survival between the W&W population and patients who underwent nCRT with resection, the chance of local regrowth is higher in the W&W population [40]. Although not explicitly mentioned by patients in this study, this may induce anxiety during the programme, especially in the first 2 years.

The interviews provided insight into the different ways of coping by colorectal cancer patients (e.g. acceptance, support by clinicians, social support, positive reappraisal and optimism). It is known that such an active coping strategy is associated with posttraumatic growth [41]. Posttraumatic growth or benefit is defined as experiencing positive psychological changes following trauma or life crises (e.g. cancer diagnosis) [42]. The different ways of coping can explain why patients feel so little hindrance in daily life. This is a common finding in cancer patients at all different stages of disease [43]. As shown by different studies conducted in breast cancer survivors, an active coping strategy improves QoL regardless of how the patient was treated [44, 45, 46].

While we have presented the identified outcomes as separate themes, all themes were interrelated. All symptoms experienced after cancer treatment have a direct and indirect impact on daily life and thereby influence the QoL of CRC patients. As one patient said: ‘When I have an appointment at ten in the morning, I need to get up at 07.30. After I get out of bed, about 15 min later, I am bound to go to the toilet. And not once, but at least three times. That has an impact on my daily life.’

Some limitations of this qualitative study need to be addressed. Firstly, it is difficult to generalize our findings to a larger W&W population; however, the findings of this study do give a general understanding of problems experienced by W&W patients. By interviewing 18 patients, with varying characteristics, and reaching data saturation after the 15th interview, we are confident that we have been able to provide a comprehensive account of the experiences of W&W patients. Secondly, telephone interviews may have influenced patients’ responses or the researcher’s interpretation of answers, because of their impersonal nature. On the other hand, patients possibly felt more comfortable being in the safe environment of their own home, which may have encouraged them to be able to open up about their problems. Lastly, the majority of patients in this study population were male, and there may be gender differences in physical symptoms, emotional burden and coping styles. By using a purposive sampling technique, a representation of the W&W population was aimed for. While women make up approximately 30% of the W&W population, they were unfortunately underrepresented in our study [2] An equal number of women and men were invited to participate, but most women declined or did not respond. The possibility exists that some issues that are important to women were therefore missed. Furthermore, there is a possibility that female participants did not feel comfortable about elaborating on certain topics regarding physical dysfunction (e.g. sexual) with a male interviewer. Another limitation could be that the patients in this study who started the W&W programme 3–5 years ago may have received limited or different information regarding potential long‐term outcomes, since these were not yet known, compared with patients who recently entered the programme. This precounselling may have affected their experience.

Most clinical studies have focused on colorectal cancer patients who, besides nCRT, underwent surgical resection, making comparison of our study results with other findings difficult. The key aspect of the W&W policy is the organ‐preserving treatment modality. To our knowledge this is the first study to investigate patients’ experiences after organ‐preserving treatment using qualitative methods. While this study suggests that patients indeed experience anxiety during the W&W programme, especially around check‐ups, a qualitative study cannot identify whether this is significantly different from the general CRC population.

The findings of this study will be used to inform the development of a novel PROM for W&W patients. Together with outcomes identified in the literature, the outcomes expressed by patients in this qualitative study will inform a longlist of potential outcomes to be incorporated in a PROM. With patient representatives and a team of experts from the International Watch‐and‐Wait Database collaboration we have set up an international Delphi study involving a range of stakeholders to reach consensus on the issues and outcomes to be addressed in the PROM [47]. With the possibility of underrepresentation of female participants in this study, we will strive for equal participation of female and male patients in the Delphi study to ensure both perspectives are included in the final PROM.

In conclusion, through direct engagement with patients in the form of individual interviews, this study provides a deeper insight into important outcomes, potential unmet needs and coping strategies in patients enrolled in our W&W programme. The findings of this study lay the groundwork for future research to develop a PROM and improve medical care for this specific patient group.

## CONFLICT OF INTEREST

We declare no conflicts of interest related to this article.

## AUTHOR CONTRIBUTIONS

Alexander J. Pennings, Merel L. Kimman and Stephanie O. Breukink were responsible for design of the study, Alexander J. Pennings collected the data, Alexander J. Pennings and Anke H. C. Gielen performed data analysis and interpretation, Alexander J. Pennings drafted the article and all authors were involved in critical revision of the article and final approval of the version to be published.

## ETHICAL APPROVAL

This study is approved by the Medical Ethical Committee of the Maastricht University Medical Centre+, the Netherlands (METC‐2019–1245).

## PATIENT CONSENT STATEMENT

All study participants, or their legal guardian, provided informed written consent prior to study enrolment.

## Data Availability

The data that support the findings of this study are available from the corresponding author upon reasonable request.

## APPENDIX 1

### PATIENT INTERVIEWS: WAIT‐AND‐SEE QUESTIONNAIRES

#### Participants

Participants will be purposively drawn from a prospectively maintained database of rectal cancer patients followed in a wait‐and‐see approach at MUMC+ and the Antoni van Leeuwenhoek Hospital. A purposive approach to sampling has been selected with the aim of maximizing diversity within the study participants. Criteria have been selected to ensure that subsets within the study population that may express contrasting views and experiences are present. These criteria for difference will be used to populate a sampling matrix.

#### Sampling matrix

Key criteria for identifying difference will include:

• Date of birth
• Gender
• Ethnicity
• Age at diagnosis
• Primary staging
  TNM classification
• Type of neoadjuvant therapy
• Interval between neoadjuvant therapy and restaging
• Duration of wait‐and‐see period (3–6 months, 12–36 months, 36–60 months and after 60 months)

#### Patient interview

During patient interview the patients’ experiences regarding subjective wellbeing and functional aspects will be explored. A semistructured format will ensure a patient‐led discussion, guided by additional prompts from a pre‐prepared topic guide to ensure key areas are covered. The topic guide may be modified during the series of patient interviews to ensure inclusion of items that have been raised by earlier participants but were not included in the topic guide.

#### Interview guide

- Participant number:
- Date of birth:
- Gender:
- Ethnicity:
- Age at diagnosis:
- Primary staging:
- Neoadjuvant treatment:
- Duration of wait‐and‐see period:

##### 1. Introduction

- Go over the purpose of the study
- Check willingness to take part in the study
- Check if the participant agrees with the interview being audio‐recorded, stored and used for presenting research findings
- Check if there are any questions regarding the interview procedure
- Ask the participant to complete the informed consent form

##### 2. Patients’ experiences with the initial treatment before the wait‐and‐see approach

1. **Ask about initial treatment for the rectal cancer**
  - How did you experience the treatment?
  - Did you experience any negative effects right after the initial treatment?
  - Do you still experience any negative effects? (Potentially provide an example such as sexual dysfunction, incontinence, etc.)
  - How did the treatment impact on your daily life?
  - What do you feel was the worst aspect of the treatment?
  - What do you feel was the most positive aspect of the treatment?

##### 3. Patients’ experiences with the wait‐and‐see treatment and the problems they encountered during this treatment

1. **Ask about the experience with the wait‐and‐see treatment (main question and potential sub‐questions)**
  - What has your experience been so far? How do you feel about this approach?
    - How do you feel about the frequency of hospital visits?
    - Do you experience any negative impacts of the watch and wait approach?
    - Are you happy with this treatment?
2. **Ask about concerns for the future**
  - Do you have any concerns for the future?
3. **Ask about what the patient thinks are important outcomes in the wait‐and‐see treatment and why (main question and potential sub‐questions)**
  - In the future we want to be able to evaluate the most important functional outcomes (i.e. anxiety, faecal incontinence, urgency) and aspects of the watch‐and‐wait approach to get a better understanding of your wellbeing and health status. Based on your experience what aspects and outcomes of the approach would be necessary to discuss with your doctor?
    - To make sure nothing is missed, due to for example shame, ask about common complaints in patients operated on for colorectal cancer (sexual dysfunction, LUTS, anxiety, incontinence)

##### 4. Closing

1. **Ask whether there is anything else they would like to talk about**
2. **Check if there are any questions**
